# Pitfalls in PSMA-PET/CT: intensive bone marrow uptake in a case with polycythemia vera

**DOI:** 10.1007/s00259-020-05072-7

**Published:** 2020-10-28

**Authors:** Philipp E. Hartrampf, Bernhard Petritsch, Andreas K. Buck, Sebastian E. Serfling

**Affiliations:** 1 Department of Nuclear Medicine, University Hospital Würzburg, Würzburg, Germany; 2 Department of Diagnostic and Interventional Radiology, University Hospital Würzburg, Würzburg, Germany

A patient with Gleason 6 prostate carcinoma (initial diagnosis April 2012) underwent [^18^F]PSMA 1007 PET/CT for restaging. After several years of watch and wait, bone metastases were detected in November 2017 with bone scintigraphy (oligometastatic disease), and anti-androgenic therapy was initiated. PSA values initially dropped, then after significant PSA relapse in March 2019 (PSA value 153 ng/ml), a second-line antihormonal therapy with abiraterone was started. Restaging with bone scintigraphy and MRI in June 2020 revealed contradictory findings. Whereas MRI findings suggested multifocal disease, the bone scan still indicated oligometastatic bone disease. For clarification, [^18^F]PSMA 1007 PET/CT was performed. PSA value at the time of imaging was 0.2 ng/ml. Additionally, the patient has been suffering from polycythemia vera (PV) for about 20 years, which is currently under therapy with hydroxyurea since December 2019. Splenectomy has been performed already in September 2019. [^18^F]PSMA 1007 PET/CT showed intense tracer uptake in the bone marrow compartment of the entire skeleton with few focal spots (A). A small accessory spleen also showed an intensive tracer uptake (A, black arrow). T1-weighted MRI of the vertebral column revealed a signal loss in vertebral bodies to the level of spinal discs, indicating a replacement of fatty bone marrow by the red bone marrow (B). Metastatic thoracic vertebra 11 presents without tracer uptake (B, C, blue arrows). Furthermore, CT revealed multiple osteoblastic lesions in the entire skeleton but without relevant tracer uptake (D, E, red arrows), indicating disease control or non-viable bone metastases under current antihormonal therapy with abiraterone, as shown recently by Plouznikoff et al. for patients receiving novel antiandrogen drugs [1]**.** In conclusion, increased [^18^F]PSMA 1007 uptake in the bone marrow compartment is most likely caused by PV, rather than bone metastases from prostate cancer.

Literature research revealed a sole case report with only mild diffuse bone marrow [^18^F]PSMA uptake in a patient with polycythemia rubra vera [2]. In our patient, [^18^F]PSMA 1007 uptake in the bone marrow compartment could be caused by the onset of myelofibrosis (MF), as the patient has been suffering from PV for several years before. Increased vascularity is assumed in PV and especially in MF [3]. Lundberg et al. have shown that myeloproliferative diseases, such as PV and MF in particular, show activation of neoangiogenesis [4]**.** Since PSMA is also expressed in the neovasculature of some tumors other than prostate cancer [5], binding to the neovasculature could occur in PV or MF, leading to non-specific tracer uptake in the bone marrow.

Our case demonstrates that [^18^F]PSMA 1007 uptake in the bone marrow can be very intense in PV, a finding that may be misinterpreted as diffuse bone metastases from prostate cancer. Although PV is a rare disease, detailed medical history is important for differential diagnosis of intense [^18^F]PSMA 1007 uptake in the bone marrow. Especially at very low PSA values or PSA values inadequate for extensive disease, one should consider the presence of hematological diseases as differential diagnosis.


